# Risk‐aware autonomous search and rescue with multiagent reinforcement learning

**DOI:** 10.1111/risa.70067

**Published:** 2025-07-05

**Authors:** Aowabin Rahman, Salman Shuvo, Samrat Chatterjee, Mahantesh Halappanavar, Terje Aven

**Affiliations:** ^1^ Optimization and Control Group Pacific Northwest National Laboratory Richland Washington USA; ^2^ Data Sciences and Machine Intelligence Group Pacific Northwest National Laboratory Richland Washington USA; ^3^ Risk Science University of Stavanger Stavanger Norway

**Keywords:** multiagent reinforcement learning, risk‐aware autonomous navigation, search and rescue

## Abstract

Autonomous navigation in dynamic high‐consequence environments, such as search and rescue (SAR) missions, often relies on multiagent robotic systems that need to learn and adapt to changing conditions. Adversarial risks can introduce further challenges in such a setting where an autonomous agent may exhibit deviations in their learned actions from training to testing. Moreover, the uncertain environment itself may also evolve with additional obstacles that can emerge during testing compared to conditions when algorithmic training of autonomous agents was performed. In this paper, we first focus on mathematically formulating the autonomous SAR problem via a risk‐aware multiagent reinforcement learning approach. Thereafter, we design and implement numerical experiments to evaluate our approach under diverse hazard scenarios with a centralized training and decentralized testing paradigm. Finally, we discuss our results and steps for further research.

## 1 INTRODUCTION

Autonomous multiagent systems (MAS) are increasingly being investigated in research for cooperative tasks in dynamic high‐consequence environments, such as during emergency response missions (Queralta et al., 2020). In these cooperative tasks, autonomous MAS can reduce overall mission time (Banas et al., 2016; Iqbal & Sha, 2019), monitor hazardous environments or events (Sung, 2019), and provide real‐time localization and mapping. One such critical task is search and rescue (SAR), where the goal is to locate a missing object/person (i.e., a “target”) of interest within a domain in the shortest possible time, where the precise location of the target may be unknown or uncertain (Banas et al., 2016). A cooperative team of autonomous robots can perform SAR tasks more efficiently by dividing the domain to perform coordinated exploration, with each agent communicating information to other agents instead of the autonomous robots independently exploring the entire region. The SAR scenario can manifest in various environments, and as such, the agents in MAS could be unmanned aerial vehicles (UAVs), unmanned ground vehicles (UGVs), or unmanned surface vehicles (USVs) (Queralta et al., 2020).

Broadly, an MAS can be defined as a system of interconnected autonomous computing entities (or “agents”) seeking to achieve a common goal (Balaji & Srinivasan, 2010). The command and control of each agent can be through a centralized or decentralized approach (Ota, 2006). The key advantage of using an MAS is that agents working in parallel can likely accomplish a task in a timely manner (Gonzalez et al., 2007). Distributing tasks with communication between the agents also reduces the need for onboard data processing and storage (Nathan et al., 2011). Deploying an MAS also avoids overdependence on a single agent and increases the probability of completing a task in the event one or more of the agents are damaged or compromised (Nathan et al., 2011). In addition, using an MAS‐based approach enables the system to be modular, allowing for flexibility to scale up as needed (Balaji & Srinivasan, 2010). In this paper, we are concerned with an MAS of autonomous vehicles that can be categorized as a cyber–physical system (CPS). A CPS can be characterized by the presence of a physical layer, that is, a network of sensors and actuators, and a cyber layer, that is, control algorithms and communication protocols.

However, a CPS can be vulnerable to several types of risks and threats—for instance, network‐enabled information transfer between cooperative agents can leave CPS exposed to interference from external entities, which makes secure control of CPS of critical importance (He et al., 2021). Interference from malicious agents can disrupt the operational goals of CPS, including closed‐loop stability, safety, and performance. Previous work (Audigier et al., 2000; Baker & Allin Cornell, 2005; Moehle & Deierlein, 2004; Porter, 2003) on performance‐based risk assessment of CPS consist of the following interconnected computational components: (i) systems modeling; (ii) hazard intensity analysis; (iii) engineering parameter analysis; (iv) damage analysis; and (v) loss exceedance analysis. Brief details on each component can be found in other literature (Du et al., 2023). Specifically, loss exceedance analysis involves the probabilistic estimation of system impacts (e.g., deterioration in system performance) based on damage measures due to one or more adversarial events. Estimating losses and subsequently developing decision support capabilities can be challenging due to uncertainties arising from unknown or complex dynamics of the environment (Du et al., 2023) and/or lack of knowledge about the future states of the CPS, but is critical for robust and secure control of CPS.

As discussed in Du et al. (2023), the decision support for CPS can be mathematically formulated as a sequential decision‐making (SDM) problem. An SDM is a class of problems where an agent interacts with a dynamic environment and selects actions, often under uncertainty, based on their knowledge of the environment (Russell & Norvig, 2016). Typically, an SDM problem is expressed as a Markov decision process (MDP). In an MDP, an agent selects an action at a given state, where the states of the agents evolve based on their actions in a probabilistic manner. One critical assumption in an MDP setting is that the next state of an agent depends on its current state and action only and not on the past trajectory of states that the agent experienced (Kochenderfer, 2015). Reinforcement learning (RL), a branch of machine learning applicable in SDM settings via agent–environment interactions, is often used to solve an MDP problem  (Sutton & Barto, 2018). RL is an optimization‐based approach where an agent computes an optimal policy (i.e., a state‐action mapping) to accomplish their objectives  (Kaelbling et al., 1996; Russell & Norvig, 2016; Sutton & Barto, 2018). In an RL context, an optimal action seeks to maximize immediate returns (i.e., a measure of how well an agent performs at a given timestep) and expected future returns. RL algorithms are typically data‐driven, where an agent learns an optimal policy through repeated interactions with an environment.

RL has gained traction in autonomous navigation tasks, including SAR. Traditionally, frontier‐based exploration algorithms have been leveraged for SAR tasks (Doroodgar et al., 2014; Mei et al., 2006; Rahman et al., 2022; Yamauchi, 1997), which are based on the concept that autonomous agents exploring the boundary between the known region and the unchartered region will lead to learning new information about the environment (Yamauchi, 1997). However, these frontier‐based approaches typically require topological information about the environment a priori. As such, it can be advantageous to use RL algorithms that do not require the layout of the environment a priori or if the environment has changed significantly over time, for example, during postdisaster navigation (Casper & Murphy, 2003). Despite advantages of using RL for SAR tasks, from a risk perspective there remains a critical research gap where multiple hazard events that could adversely impact a system need to be addressed while determining optimal navigation control policies. In the case of SAR, autonomous agent performance may be susceptible to interference from a malicious adversary  (Rahman et al., 2022) as well as presence of physical hazards within a dynamic environment  (Iqbal & Sha, 2019). The core research need that is addressed in this paper pertains to developing RL algorithms that simultaneously address coupled hazard events arising from both adversarial interference and disruptive environmental changes.

Based on this critical need, we focus on two key research questions: (i) how to quantify the coupled impact of risks from effects of a malicious adversary and physical hazards in an environment? and (ii) how to develop a multiagent RL (MARL) algorithm robust to adversarial interference to minimize impacts from these risks? Our novel contributions to address these research questions within a risk‐aware MARL context are:

- Advancing an agent's state estimation by incorporating local awareness of hazards in neighboring regions.
- Developing a composite reward structure to minimize coupled effects of adversarial and environmental hazards.

The rest of the paper is organized as follows. Section 2 presents a risk framework used in this paper. In Section 3, we present the mathematical formulation of the SAR problem. Section 4 describes the risk‐aware MARL algorithmic methodology to solve the SAR problem. Numerical experiments and results are presented in Section 5. Finally, we discuss conclusions and steps for further research in Section 6.

## 2 RISK FRAMEWORK

This section describes the risk framework used in this paper for addressing the SAR problem within an autonomous MAS context. Our problem framing is consistent with the risk triplet elements including uncertainty (Kaplan & Garrick, 1981) and probabilistic principles for evaluating safety of engineered systems (Apostolakis, 1990, 2004; Bier & Lin, 2013; Haimes, 2009; Paté‐Cornell, 1996). In an SAR context with autonomous MAS, *safety* may refer to structural integrity of agents based on continual operations in a hazardous environment, *security* may refer to adversarial interventions that could lead to undesirable impacts to mission success, and *severity* may refer to consequences of adverse events that may be characterized by deviations to mission performance. We explore multiple failure scenarios accounting for safety and security considerations (e.g., with obstacles, pitfalls, and adversarial agents in a navigation environment), and assess SAR mission performance via multiple severity metrics (e.g., target retrieval time and success rate). Moreover, we leverage contemporary risk science knowledge (Aven, 2012, 2016; Society for Risk Analysis, 2018a, 2018b), where risk as a concept is related to an activity comprised of two main components: consequences (or reformulated as events and related consequences), and associated uncertainties (i.e., what will the consequences be?). We focus on negative or undesirable consequences, and risk as a concept is then intuitively reflecting that such consequences may occur. In a risk assessment, risk is measured or characterized by specifying the consequences (events and consequences), and using a measure or description to express the related uncertainties. In its most general form, such a risk description takes the form (A, C, Q, K), where A denotes the events, C the related consequences, Q is a description of the uncertainties about A and C, and K is the knowledge supporting the judgments of Q, A, and C. Typically Q is represented by knowledge‐based (subjective) probabilities P. It is also common in this setup to refer to the risk sources, RSs. A risk source RS is defined as “an element (i.e., action, subactivity, component, system, or event), which alone or in combination with other elements has the potential to give rise to some specified consequences (typically undesirable consequences)” (Society for Risk Analysis, 2018a). Hazards and threats can be viewed as risk sources. A hazard is defined as a risk source where the potential consequences relate to harm, whereas a threat is a stated or inferred intention from an adversary to inflict harm. Threats can manifest themselves in hazards or environmental damage, or direct hindering of the mission.

An autonomous MAS setting for SAR involves multiple decision agents interacting with an uncertain navigable environment to determine optimal paths to and from objects of interest. Hazards in this setting may involve physical barriers that prevent navigation or adversarial interventions such as communication of misinformation about the system with malicious intent. Real‐world risk analyses with deep uncertainties, such as SAR with autonomous MAS, may involve system state probabilities that can be challenging to quantify. An alternative to probabilistically modeling a system first and then determining optimal actions that mitigate hazards is to directly learn a mapping between system states and actions based on autonomous agent interactions with an environment (Cox, 2020). From a mathematical problem formulation standpoint, SDMs may include probabilistic representation of transition between system states, as well as belief state distributions over states representing system uncertainties. However, from an algorithmic and computational standpoint, solving such SDMs rely on deep RL models that inherently account for system uncertainties through data captured while interacting with the environment during training; without the explicit need for assigning probability values. Recent examples where deep RL approaches have been applied for risk and decision analysis include robust control of cyber–physical energy systems (Du et al., 2023) and autonomous path planning for disaster management (Li & Wang, 2025).

Given this risk backdrop, a partially‐observable MDP (POMDP) formulation is presented in this paper. POMDPs provide a method for optimizing the rewards and minimizing the consequences over a specified period of time while accounting for uncertainty in future actions and consequences. This formulation is based on the following elements:

S: set of system states;
A: set of possible actions;
R: set of rewards associated with state and action combinations;
$P_{a}=P\left(s^{\prime }|s,a\right)$: transition probabilities between states $s\to s^{\prime }$ due to action a;
γ: discount factor for cumulative rewards;
O: set of observations for all agents;
Z(o|s,a): observation distribution after action a;
b(s): belief state distribution; and
K: supporting knowledge.

The system state S reflects the different events A and aspects of C. The C also captures the rewards R. Action set A refers to the possible decisions that can be taken. The uncertainties in system states are reflected by $P_{a}$, Z(o|s,a), and b(s); whereas value reductions for future rewards accumulated due to a given action is accounted for using the discount factor γ. Z(o|s,a) denotes the probability distribution associated with an observation o due to an action a at a given state s. Since the joint state is not fully observed, a belief state distribution b(s) describes the probability of the environment being in state s and is iteratively computed as the state transitions to $s^{\prime }$ based on action a and new observation o as:

(1) $$b\left(s^{\prime }\right)=\eta Z\left(o|s^{\prime },a\right)\sum_{s\in S}P\left(s^{\prime }|s,a\right)b\left(s\right).$$

Here, η is a normalizing constant defined as $1/[\sum_{s^{\prime }\in S}Z\left(o|s^{\prime },a\right)\sum_{s\in S}P\left(s^{\prime }|s,a\right)b\left(s\right)]$. The knowledge K specifically captures the modeling assumptions.

In an autonomous MAS setting (e.g., SAR problem in this paper), environmental and adversarial risks can interfere with cooperative mission success. Sources of uncertainty could include a lack of knowledge about the environment and limited capability to communicate with other agents in a cooperative mission. As described in Section 1, RL is often used as an algorithmic approach for tasks related to autonomous navigation formulated as an MDP. RL involves an agent learning an optimal policy to make sequential decisions in an environment (Sutton & Barto, 2018), not only to maximize immediate gains (or rewards), but also maximize cumulative gains over the long term. This makes RL particularly suited for planning tasks which can involve risk‐informed decision support. Thus, we leverage RL as a quantitative approach to minimize consequences over the long term while accounting for uncertainty in future actions and consequences.

## 3 PROBLEM FORMULATION

**FIGURE 1:**
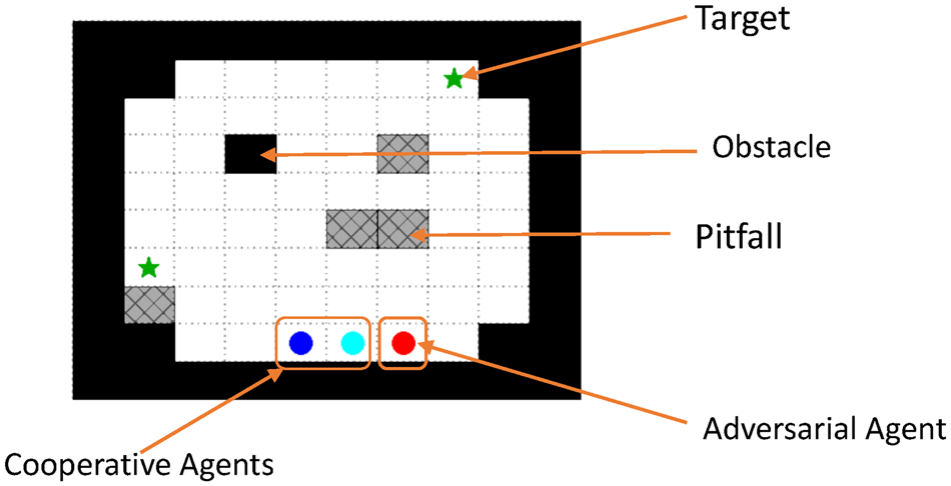
Illustration of the search and rescue (SAR) problem setup: the goal of the cooperative agents is to reach the target locations in the shortest possible time. Two sources of risks are presented here: (A) adversarial agents trained as cooperative agents acting to derail other cooperative agents during testing and (B) pitfalls in an environment that can lead to termination of an agent navigation sequence. Note that the *Pitfalls* are different than the *Obstacles*. *Obstacles* are grid cells that an agent cannot occupy and are forced to move around during navigation; *Pitfalls* are grid cells that terminate a navigation sequence once an agent moves into them.

We consider an SAR problem setup where we have a cooperative team of N agents in a gridworld environment (Goel et al., 2021), with the goal of identifying one or more missing objects/persons (i.e., “targets”) within the domain in the shortest possible time (see Figure 1). The location of the targets are not known a priori to the cooperative agents. Two sources of risk arise: (i) after all the agents are deployed as part of the cooperative team, one or more agents exhibit adversarial behavior (possibly due to external adversary interference) to derail the cooperative mission and (ii) the environment can have *pitfalls* (i.e., locations not known to the cooperative agents a priori) that can halt the navigation progress of cooperative agents and terminate the SAR mission. Please note that *pitfalls* are different from *obstacles*. Pitfalls are grid cells that terminate a navigation sequence once an agent moves into them. For example, in a real‐world navigation context under uncertainty, pitfalls may represent a land mine which may terminate the navigation exercise completely if an agent steps on it. On the other hand, obstacles are grid cells that an agent cannot occupy and are forced to move around during navigation. In a navigation setting, an obstacle may represent a physical barrier (e.g., debris, concrete block) that does not terminate the navigation exercise if an agent steps on it but forces the agent to maneuver their way around while continuing their navigation task.

Key assumptions within this SAR problem setup are as follows:

- All agents have homogeneous sensing capabilities.
- All agents have shared understanding of the navigation progress of other agents during centralized training.
- During decentralized execution, cooperative agents aim to optimally explore the search region.
- The adversarial agent misleads the cooperative agents about the target locations.
- The target locations are static and do not change with time after the agents are deployed.
- The grid has pitfalls that represent environmental hazards.

This SAR problem with multiple agents can be mathematically formulated further as a Decentralized POMDP (Dec‐POMDP). A Dec‐POMDP typically consists of multiple computing entities (called “agents”), that interact with the environment (which may include communication with other agents) and independently take actions. In this setting, a policy π maps an action to the belief state. In a Dec‐POMDP, the goal is to solve for a set of decentralized policies $\pi =\{\pi^{1},\pi^{2}\text{…}\pi^{N}\}$ for each agent n∈N. The value function at a given timestep $V_{t}^{\pi }\left(b\right)$ for a given policy π is (Skoglund, 2021):

(2) $$\begin{array}{ccc} V_{t}^{\pi }\left(b\right) & = & {\text{argmax}}_{a\in A}\left[\sum_{s\in S}r\left(s,a\right)b\left(s\right)\right.\\ & & \left.+\;\gamma \sum_{o\in O}p\left(o|b,a\right)V_{t-1}\left(b^{o,a}\right)\right]. \end{array}$$

A Dec‐POMDP is solved by computing the optimal policy that maximizes the value function $\pi^{*}={\text{argmax}}_{\pi }V^{\pi }$. Reward r(s,a) refers to a reward realization for a particular state and action combination. In Section 2, R refers to the set of rewards associated with all state and action combinations. Exact solution to Dec‐POMDPs is often impractical or intractable. Thus, MARL algorithms which model the policies of the agents as neural networks (NNs) are often used as a practical alternative (Lee & Lee, 2021). Actor–critic methods are often used for Dec‐POMDPs as they allow the use of a centralized critic to evaluate the performance of decentralized policies in a centralized training with decentralized execution (CTDE)‐based learning framework, which has been shown to achieve benchmark performance in MARL problems, especially in cooperative and competitive environments (Srinivasan et al., 2018). In CTDE learning, the critic has access to global information about the environment during training; however, during deployment, the agents execute their policies using their own observations only (Ikeda & Shibuya, 2022). In this paper, we use a version of soft actor–critic (SAC, Haarnoja et al., 2018) as used by Iqbal and Sha (2019), as SACs promote exploration and can potentially capture multiple modes at which near‐optimal solutions are observed (Haarnoja et al., 2018). In the next section, we describe a risk‐aware MARL algorithmic methodology to solve the SAR problem.

## 4 METHODOLOGICAL APPROACH

**FIGURE 2:**
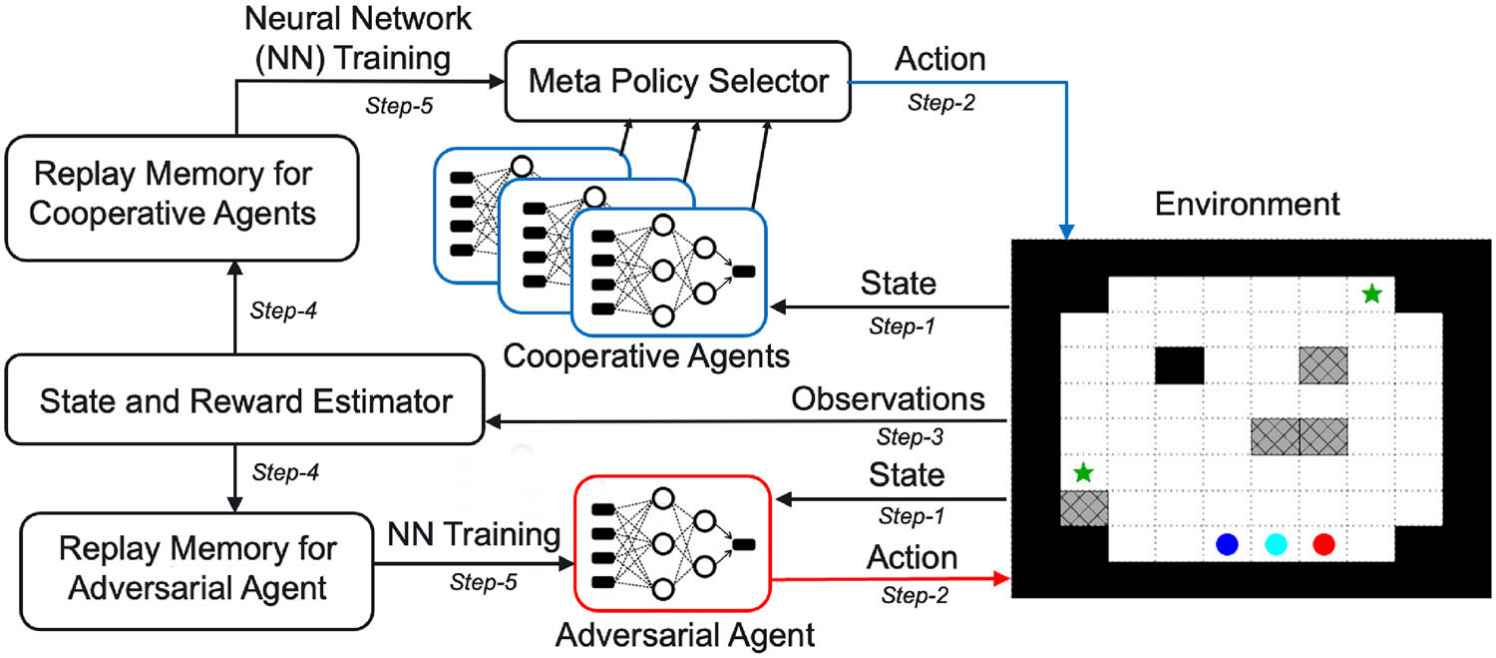
Overview of risk‐aware multiagent reinforcement learning (MARL) methodological approach. The approach depicts agent–environment interactions and a closed‐loop computational workflow where states of the environment (i.e., a grid displayed here) inform agent actions through training and subsequently trigger state transitions and realization of rewards.

Our methodology to solve the SAR problem described above involves developing a risk‐aware MARL algorithm incorporating local awareness in an agent's state estimation and a composite reward structure formulation to capture the coupled adversarial and environmental hazard effects. Figure 2 presents our overarching methodological approach, including five key steps. In step 1, both cooperative and adversarial agents receive state information with local awareness based on observations from the environment. Next, the agents compute and execute optimal actions in step 2. After that, in step 3, observations from the environment are transmitted to a state transition and composite reward estimation compute module. In step 4, state transition and reward estimates are conveyed to replay memories for cooperative and adversarial agents. The information stored in these replay memories is subsequently used for NN training of the agents in step 5, which leads to the generation of policies and updated actions over time. The adversarial agent's policy is updated after each iteration of training of the cooperative agents. Description of state estimation with local awareness, composite reward structure, and risk‐aware MARL algorithm follows.

### 4.1 State estimation with local awareness

The agent actions or decisions are based on their state observations with local awareness from the environment; thus, they do not require explicit communication with each other. This enables them to be robust from communication noise and external disturbances. The agents can access information about their neighboring regions (i.e., cells in the grid), and we capitalize on this information to assure local awareness. Also, we ensure that the state space is well‐defined for stable training with the following observations for each agent:

- Current location ($x_{i},y_{i}$)
- Existence of obstacles in each of the four surrounding cells
- Existence of pitfalls in each of the four surrounding cells
- Location of other agents if they are within a certain distance
- Number of retrieved targets

Note that we advance the state definition in Rahman et al. (2022) by incorporating whether or not each of the four surroundings cell is a pitfall as an additional set of observations for each agent. The joint observation of all agents constitutes the state space. The action space consists of moving one step in the grid to the four possible cardinal directions (i.e., north, east, south, and west) or staying in the current location for each agent, $A_{i}\in \left\{up,down,right,left,stay\right\}$. An agent rescues a target if they reach their location in the grid.

### 4.2 Composite reward structure

We model a composite reward structure addressing risks from the environment and adversaries and adapt the learning algorithms proposed in Iqbal and Sha (2019) and Rahman et al. (2022). As the cooperative and adversarial agent objectives are different, we formulate separate reward functions for them as described below.

#### 4.2.1 Cooperative agents

The cooperative agents receive a composite reward, $r^{c}$, at every timestep that includes a state novelty reward $r_{\mathrm{nov}}^{c}$, a secondary reward $r_{\sec}^{c}$, and event‐based external reward $r_{\mathrm{ext}}^{c}$. For the sake of brevity, we remove the suffix i from reward representation for the ith agent. Since an SAR mission presents a sparse‐reward challenge, where the number of missing targets is relatively small compared to the domain's size, novelty rewards play a crucial role in training agents to efficiently explore the domain. The novelty reward $r_{\mathrm{nov}}^{c}$ represents the different team collaboration strategies that the meta policy selection model capitalizes, adopted from Iqbal and Sha (2019). The novelty reward is estimated from the exploration of the agent's observations at a specific location $\left(x_{i},y_{i}\right)$ based on the number of previous visits $v_{i}$ by all agents. For a discrete environment, such as gridworld, the inverse of state visits forms a suitable novelty function, $\zeta_{i}=\frac{1}{v_{i}}$ that can be used to formulate novelty rewards to portray three distinct exploration strategies:

- The **Minimum** strategy rewards an agent when it explores a location that no other agent has visited. This novelty reward is defined as $r_{\mathrm{nov}}^{c}=\min_{j\in \{1,\text{…},N\}}\zeta_{j}$.
- The **Covering** strategy rewards an agent for exploring a location more novel than the average agent. It is defined as $r_{\mathrm{nov}}^{c}=\zeta_{i}1\left[\zeta_{i}>\bar{\zeta }\right]$, where $\bar{\zeta }=\sum_{j}\zeta_{j}/N$ and 1(·) denotes an indicator function.
- The **Burrowing** strategy, on the other hand, incentivizes agents to explore locations less novel than the average agent. This approach enables agents to quickly identify dead‐ends, potentially reducing overall exploration time. It is expressed as $r_{\mathrm{nov}}^{c}=\zeta_{i}1\left[\zeta_{i}<\bar{\zeta }\right]$.

We include a secondary reward, $r_{\sec}^{c}=\sum_{j=1}^{N}1\left[v_{j}\left(x_{i},y_{i}\right)=1\right]$ from Rahman et al. (2022) along with the novelty rewards to further enhance exploration. Furthermore, the agents receive external rewards $r_{\mathrm{ext}}^{c}$ that portray their local awareness. Finally, the composite reward for a cooperative agent is a weighted sum of the above‐mentioned rewards:

(3) $$r^{c}=r_{\mathrm{nov}}^{c}+\alpha_{1}r_{\sec}^{c}+\beta_{1}r_{\mathrm{ext}}^{c},$$

where $\alpha_{1}$ and $\beta_{1}$ are cooperative agent reward weight coefficients. The external rewards $r_{\mathrm{ext}}^{c}$ only include a penalty for falling in a pit $r_{\mathrm{pit}}$ during training. In addition, during testing, cooperative agents receive time penalties $r_{\mathrm{time}}$ for each timestep, earn rewards $r_{\mathrm{target}}$ for locating missing assets, and $r_{\mathrm{done}}$ for completing the mission (i.e., discover all targets).

#### 4.2.2 Adversarial agent

The adversarial agent's reward $r^{a}$ consists of secondary reward $r_{\sec}^{a}$ and external reward $r_{\mathrm{ext}}^{a}$. Adversarial agents are incentivized to delay the cooperative agents rather than directly locate the targets themselves. If an adversarial agent encounters a target, it provides false information about their location. The secondary reward for the adversarial agent is adopted from Rahman et al. (2022) as:

(4) $$r_{\sec}^{a}=\sum_{j=1}^{N}1\left[v_{j}\left(x_{i},y_{i}\right)>v_{\mathrm{thresh}}\right].$$

Here, $v_{\mathrm{thresh}}$ represents a threshold value above which a state visit is deemed unnecessary (e.g., $v_{\mathrm{thresh}}=1$). This enables the adversarial agents to aim to prompt cooperative agents to revisit previously explored states, thus increasing redundancy. The adversarial agents receive external rewarded $r_{\mathrm{ext}}^{a}$ for each timestep and for hindering the cooperative agents, but not for locating assets. The final reward for an adversarial agent is:

(5) $$r^{a}=\alpha_{2}r_{\sec}^{a}+\beta_{2}r_{\mathrm{ext}}^{a},$$

where $\alpha_{2}$ and $\beta_{2}$ are adversarial agent reward weight coefficients.

### 4.3 Risk‐aware MARL algorithm

Algorithm 1 depicts the risk‐aware MARL approach as a two‐tier structure (adapted from Iqbal & Sha, 2019) to enable collaboration among the cooperative agents. Below is a brief outline of this two‐tier structure:

- **Upper‐Tier Policy Selection Model**: This model termed a metapolicy model, selects a single policy from a set of policies that all agents will adopt. Each policy corresponds to a different collaboration strategy *minimum, covering or burrowing* for exploration among the cooperative agents. The upper‐tier model's parameters, denoted as ϕ, are learned through policy‐gradient updates (Iqbal & Sha, 2019).
- **Lower‐Tier SAC Model**: This model optimizes individual agent policies based on collaborative exploration strategies or novelty functions. It utilizes a central critic network shared across all agents and a policy model specific to each agent. To improve sample efficiency, the model employs off‐policy learning methods, where all policies and value functions are learned using all available data, regardless of the policy and exploration strategy used to generate the data. For both the critic and the actor networks, we use a multilayered perceptron NN with one hidden layer.

ALGORITHM 1 **Risk‐Aware MARL.**

**Table:** 

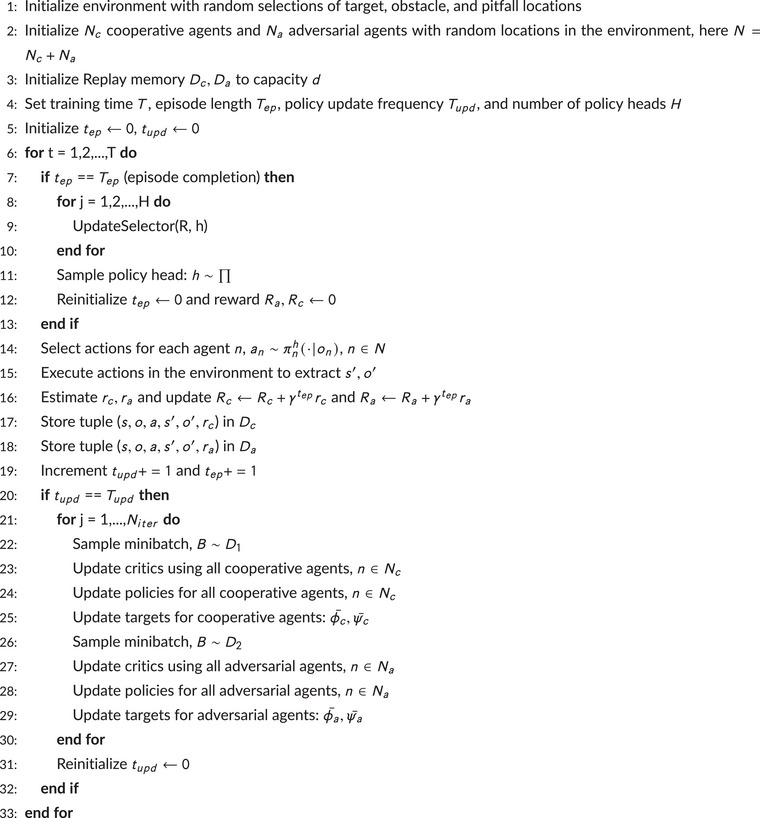

We follow the CTDE‐based learning framework where agents exchange information while training, such as observations of other agents, but operate independently based on their own local observations during execution (i.e., testing and implementation). This approach is beneficial for avoiding the issue of nonstationarity commonly encountered in training MAS. Having a centralized critic that considers observations from all agents, leads to agents being relatively less susceptible to sudden changes in the environment; leading to enhanced training stability. Algorithm 1 indicates that both a global state and reward function are utilized to train each agent's policy, necessitating access to information from other agents. However, during execution, agents only rely on their local observations and act according to their individual policies. The MARL training procedure involves alternately training the cooperative and adversarial agents. During training, both types of agents aim to maximize their own rewards, leading to the cooperative agents becoming more resilient to adversarial influences. The reward structure suggested for a general optimal‐coverage problem does not need the final state to rely on where the targets are located. So, during training, both cooperative and adversarial agents are trained without any targets on the grid. Nevertheless, during testing and execution, they revert back to the SAR scenario, where cooperative agents need to find their targets.

## 5 NUMERICAL EXPERIMENTS

To evaluate our risk‐aware MARL methodology for the SAR problem, we developed multiple simulation scenarios involving environmental and adversarial risks in a 20×20 grid (based on schematic in Figure 1). A description of these scenarios, training strategies, and testing results are discussed below.

### 5.1 Scenarios

We developed the following simulation scenarios—each containing multiple sources of risk with progressively increasing levels of complexity:

- **Scenario 1** portrays a risk‐free setting with obstacles. Two agents are tasked with collaboratively searching across a grid to retrieve targets of interest in the shortest possible time.
- **Scenario 2** presents environmental risks to the agents in the form of pitfalls along with obstacles. In addition to performing optimal search, the agents also need to avoid the pitfalls. We assume that both agents are cooperative.
- **Scenario 3** introduces a third agent in the SAR environment with obstacles, but without any pitfalls. This agent is assumed to be cooperative during training, but during testing this agent turns adversarial. The cooperative team's approach is to mitigate adversarial effects during their search.
- **Scenario 4** extends scenario 3 with the presence of pitfalls.

We compare the performance of our risk‐aware MARL algorithm with methods proposed by Iqbal and Sha (2019) (considers only environmental risks) in scenarios 2 and 4, and with Rahman et al. (2022) (considers only adversarial risks) in scenarios 3 and 4. The desired outcome of this comparative analysis was to determine if our algorithm led to comparable or even better results than the current state of the art.

### 5.2 Training

**FIGURE 3:**
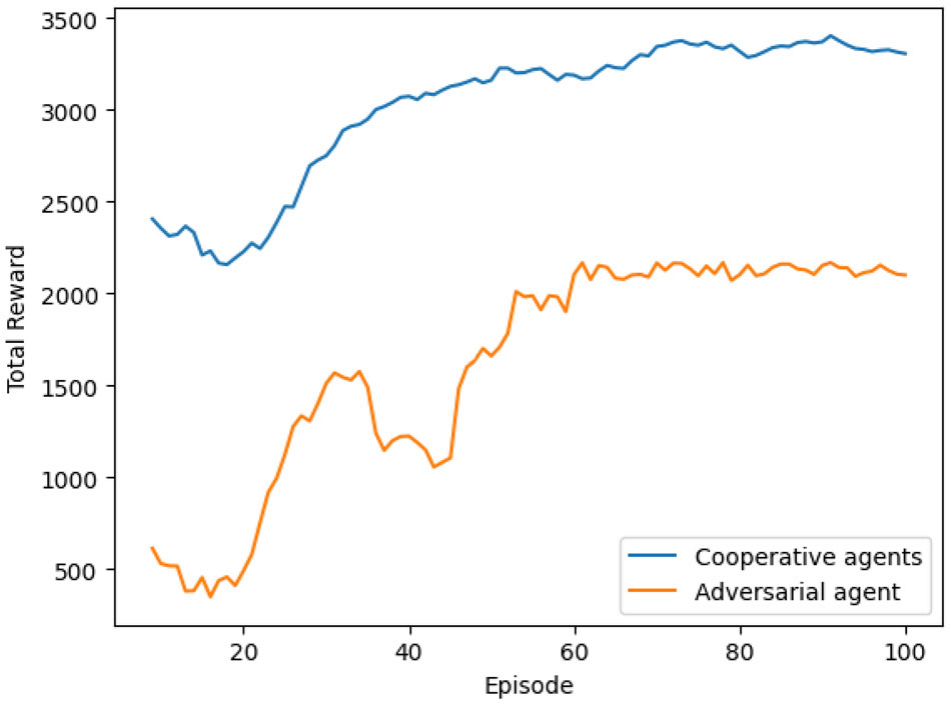
Episodic rewards accumulated by cooperative and adversarial agents in training with the 20×20 grid under scenario 4. Cooperative and adversarial agent rewards per episode increase with time and converge approximately after 60 episodes.

For risk‐aware MARL algorithmic training, within a 20×20 grid, we assume 5% of grid cells as *pitfalls* and 7% of grid cells as *obstacles*. The extent of pitfalls and obstacles in the grid were inspired by gridworld settings in Iqbal and Sha (2019) and Rahman et al. (2022), respectively. For the composite reward function (see Equations 3– 5) based on optimal coverage, we train the agents without the target locations. A training episode is defined as a sequence of steps taken by each learning agent starting from their initial location in the grid for target retrieval. Each episode terminates if an agent steps into a pitfall or the maximum exploration of 500 timesteps is reached, whichever occurs sooner. In this paper, a simulation timestep is defined as an instance where a set of actions are taken by the agents concurrently. Figure 3 presents the total accumulated reward (smoothed based on the moving average of last 10 episodes) for the cooperative and adversarial agents in scenario 4. We observed similar training patterns for all other scenarios (not displayed here for the sake of brevity). Agent reward per episode increases over time as they learn, and flattens after several episodes, representing training convergence. We terminate the training if the agents cannot increase their maximum episodic reward over 20 episodes. The 20×20 grid required approximately 60 episodes to converge.

Table 1 summarizes the hyperparameters used across all scenarios in the simulation experiments. These experiments were performed using high‐performance computing with AMD EPYC 7502, 2.5 GHz CPU, and 256 GB RAM. The risk‐aware MARL algorithm was implemented in Python 3.7.3 with VS Code Version: 1.85.2 (Universal). The convergence clock times for the risk‐aware MARL algorithm across the four simulated scenarios were 26 h (scenario 1), 28 h (scenario 2), 55 h (scenario 3), and 60 h (scenario 4). These convergence clock times were obtained via parallelization of simulation runs across 12 CPU cores. Further reduction in convergence times can be achieved with model training using GPUs; however, that is an expensive compute option and was outside the scope of this study.

**TABLE 1:** Hyperparameter settings across all scenarios in the simulation experiments.

Name	Description	Value
$Q_{lr}$	Learning rate for centralized critic	0.001
$Q_{optimizer}$	Optimizer for centralized critic	Adam (Kingma & Ba, 2015)
$\pi_{lr}$	Learning rate for decentralized policies	0.001
$\pi_{optimizer}$	Optimizer for decentralized policies	Adam
$\Pi_{lr}$	Learning rate for policy selector	0.04
$\Pi_{optimizer}$	Optimizer for policy selector	SGD (Amari, 1993)
τ	Target function update rate	0.005
bs	Batch size	1024
Total steps	Number of total environment steps	$1\times 10^{6}$
Steps per update	Number of environment steps between updates	100
Niters‐model	Number of iterations per update for policies and critics	50
Max ep length	Maximum length of an episode before resetting	5000
Ψ	Penalty coefficient for weight decay on parameters of Q‐function	0.001
Θ	Penalty coefficient on L2 penalty on pre‐softmax output of policies	0.001
θ	Penalty coefficient for weight decay on parameters of policy selector	0.001
|D|	Maximum size of replay buffer	$1\times 10^{6}$
γ	Discount factor	0.99
α	Weight coefficient of secondary reward	0.1
β	Weight coefficient of external reward	1
ζ	Decay rate of count‐based rewards	0.7

### 5.3 Testing results and discussion

In this section, we present results from our numerical experiments based on the four scenarios defined above. During testing, we compare the performance of our risk‐aware MARL algorithm against two baseline algorithms (Iqbal & Sha, 2019; Rahman et al., 2022) with different environmental configurations than training (i.e., obstacles and pitfalls are at different locations within the grid compared to training; and two SAR targets are introduced at different locations within the grid). The first step for evaluating our risk‐aware MARL algorithm with state‐of‐the‐art methods is defining a performance metric—*retrieval time*—expressed as average time over episodes that the cooperative team takes to retrieve all targets. Another performance metric we evaluated is the *success rate* under time and resource constraints, representing the percentage of successful rescues of targets within a fixed number of timesteps. Time and resource constraints are critical considerations in SAR operations, as they directly impact the efficiency, effectiveness, and outcome of the mission. Search vehicles, drones, or necessary data feeds like thermal imaging cameras typically have limited operational time due to fuel or battery constraints.

**FIGURE 4:**
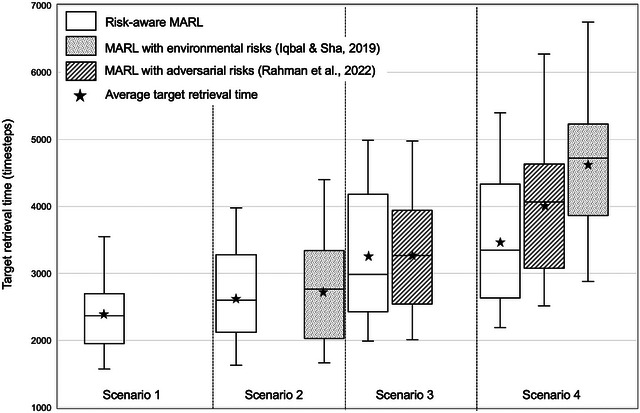
Variability in target retrieval times over multiple simulation episodes for risk‐aware multiagent reinforcement learning (MARL) algorithm and other methods across scenarios.

Figure 4 displays variability in target retrieval times over multiple simulation episodes for risk‐aware MARL algorithm and other methods across four scenarios. The average and median target retrieval times based on the risk‐aware MARL algorithm increase with increasing complexity of scenarios from 1 to 4. As described in Section 4, in scenarios 3 and 4, the training accounts for an adversary; both training and testing were performed with two cooperative agents and one adversarial agent. Scenario 1 is the fastest SAR task with an average retrieval time of 2408 timesteps and a median retrieval time of 2370 timesteps. Including pitfalls in the environment (scenario 2) increase the retrieval times based on risk‐aware MARL algorithm by approximately 9% to 2650 timesteps (average) and 2601 timesteps (median) compared to scenario 1, as agents must adjust their paths to avoid obstacles. However, in scenario 2, our risk‐aware MARL algorithm achieved a retrieval time that was 3% lower than the 2740 timesteps (average) and 6% lower than the 2764 timesteps (median) observed in MARL with environmental risks (Iqbal & Sha, 2019). In scenario 3, which includes an adversarial agent, the cooperative agents take longer to retrieve the targets based on risk‐aware MARL algorithm by approximately 36% to 3275 timesteps (average) and 26% to 2984 timesteps (median) compared to scenario 1. However, our risk‐aware MARL algorithm achieved a retrieval time that was 0.4% lower than the 3288 timesteps (average) and 9% lower than the 3266 timesteps (median) observed in MARL with adversarial risks (Rahman et al., 2022). Finally, in scenario 4, we observe that the retrieval time using our risk‐aware MARL algorithm increases by 47% to 3546 timesteps (average) and 41% to 3347 timesteps (median) when compared with scenario 1. We also observe that in scenario 4, our risk‐aware MARL algorithm performs marginally better than both Iqbal and Sha (2019) (with 23% reduction in average retrieval time and 29% reduction in median retrieval time) and Rahman et al. (2022) (with an 11% reduction in average retrieval time and 18% reduction in median retrieval time) due to our composite reward structure and state definition. The method in Iqbal and Sha (2019) yields the longest retrieval time as it does not incorporate adversarial presence during training and indicates that the coupled effects of environmental hazards and malicious adversary can be significant. These observations above suggest that while coupled effects of environmental and adversarial risks can hinder the performance of cooperative agents (i.e., increase retrieval time), incorporating them as part of training can ensure better testing performance compared to cases without risks during training. The standard deviation in retrieval times across different methods are comparable (within 9%) in the most complex scenario 4. Moreover, risk‐aware MARL algorithm has the least standard deviation among all methods in scenario 4. Overall, our risk‐aware MARL algorithm performs marginally better than state‐of‐the‐art algorithms across scenarios 2 and 3; and significantly better than state‐of‐the‐art MARL algorithms in the most complex scenario 4, based on reduced times required to retrieve all the targets from the environment.

Next, we discuss statistical significance test results comparing retrieval times based on the risk‐aware MARL and other state‐of‐the‐art reference algorithms. We applied the Kolmogorov–Smirnov (K–S) test (Hodges, 1958) to evaluate statistical significance of differences in retrieval time distributions between risk‐aware MARL and other state‐of‐the‐art reference algorithms across scenarios 2, 3, and 4. The K–S test is nonparametric, used for comparing one‐dimensional continuous distributions, and is suitable for this study due to unequal sample lengths without assumptions about normality of retrieval time distributions. For each scenario, we perform a one‐tailed K–S test at 95% confidence level by considering an alternative hypothesis, $H_{a}$, that the risk‐aware MARL retrieval time ($t_{ra}$) is lower than retrieval times based on other state‐of‐the‐art reference algorithms; and only reject the null hypothesis, $H_{0}$, in favor of $H_{a}$ when the p‐value, p<0.05. For scenario 2, the reference algorithm is MARL with environmental risks; for scenario 3, it is MARL with adversarial risk; and for scenario 4, we consider both of these algorithms as references. Table 2 presents the K–S test results. We observe that only for case III under scenario 4, where we compare risk‐aware MARL against MARL with environmental risk, the risk‐aware MARL retrieval times are lower with statistical significance at 95% confidence level. Thus, even though the mean and the median retrieval times are lower for risk‐aware MARL compared to reference algorithms across scenarios 2–4, the reduction in retrieval times is only statistically significant for case III. However, it should be noted that for a given scenario, retrieval times only consider attempts by cooperative agents where the targets were retrieved within a threshold number of timesteps, and does not account for failed attempts. As such, comparing retrieval times is necessary but not sufficient to adequately analyze the performance of a given MARL algorithm, and should be considered along with another performance metric such as *success rate* (described next), which captures the fraction of successful target retrievals out of a total number of search attempts.

**TABLE 2:** Kolmogorov–Smirnov (K–S) test results at 95% confidence level comparing retrieval times for risk‐aware multiagent reinforcement learning (MARL) ($t_{ra}$) against MARL with environmental risks ($t_{env}$) and MARL with adversarial risks ($t_{adv}$) across scenarios.

Case	Scenario	Null hypothesis	Alternate hypothesis	*p*‐Value	Result
I	2	$H_{0}:t_{ra}\geq t_{adv}$	$H_{a}:t_{ra}<t_{env}$	0.511	$H_{0}$ cannot be rejected
II	3	$H_{0}:t_{ra}\geq t_{adv}$	$H_{a}:t_{ra}<t_{adv}$	0.591	$H_{0}$ cannot be rejected
**III**	**4**	$H_{0}:t_{ra}\geq t_{env}$	$H_{a}:t_{ra}<t_{env}$	**0.00684**	$H_{0}$ **rejected in favor of** $H_{a}$
IV	4	$H_{0}:t_{ra}\geq t_{adv}$	$H_{a}:t_{ra}<t_{env}$	0.108	$H_{0}$ cannot be rejected

*Note*: The bold face value for case III, scenario 4 in Table 2 indicates that the p‐value from the K‐S test is less than 0.05 and hence the null hypothesis is rejected in favor of alternate hypothesis indicating that the risk‐aware MARL retrieval time is less than MARL with environmental risk retrieval time with statistical significance at 95% confidence level.

Table 3 presents the *success rate* percentages for the agents with a maximum threshold of 3600 timesteps in simulation to finish the SAR mission. Note that we selected this maximum threshold since it just greater than the average retrieval time (in number of timesteps) in the most complex scenario 4 with risk‐aware MARL. As expected, the success rate decreases going from scenario 1 to 4—as with increasing complexity, it is more challenging for the agents to retrieve the targets within a fixed number of timesteps. The risk‐aware MARL method achieves higher percentage success rate compared to other approaches across all scenarios. Specifically, for scenario 4 with composite risk from environment and adversary, risk‐aware MARL achieves more than 57% success rate where the other methods have a success rate below 40%. This represents an improvement by a factor of >1.5 with risk‐aware MARL algorithm compared to other methods.

**TABLE 3:** Success rate percentages of retrieving all targets with a maximum of 3600 timesteps.

Method	Scenario 1	Scenario 2	Scenario 3	Scenario 4
***Risk‐aware MARL***	***95.13 %***	***91.89 %***	***63.33 %***	***57.14 %***
MARL with adversarial risks (Rahman et al., 2022)	N/A	N/A	60.00 %	37.50 %
MARL with environmental risks (Iqbal & Sha, 2019)	N/A	88.89 %	N/A	14.29 %

Abbreviation: MARL, multiagent reinforcement learning.

**FIGURE 5:**
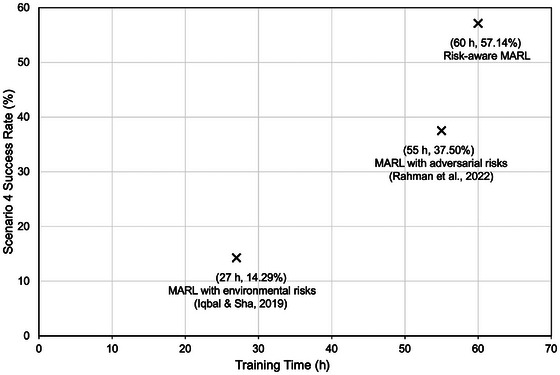
Training time and success rate trade‐off of risk‐aware multiagent reinforcement learning (MARL) algorithm and other methods for the most complex scenario 4.

Figure 5 presents a scatter plot of training time versus success rate for all methods in scenario 4. Risk‐aware MARL method achieves a relatively high success rate compared to other approaches (i.e., a margin of 20% compared to Rahman et al., 2022 and a margin of 43% compared to Iqbal & Sha, 2019). The increase in success rate comes at a cost of high training time (i.e., 9% higher training time relative to Rahman et al., 2022 and 122% higher training time relative to Iqbal & Sha, 2019). The training time and success rate trade‐off suggests that risk‐aware MARL is a feasible algorithmic option.

**FIGURE 6:**
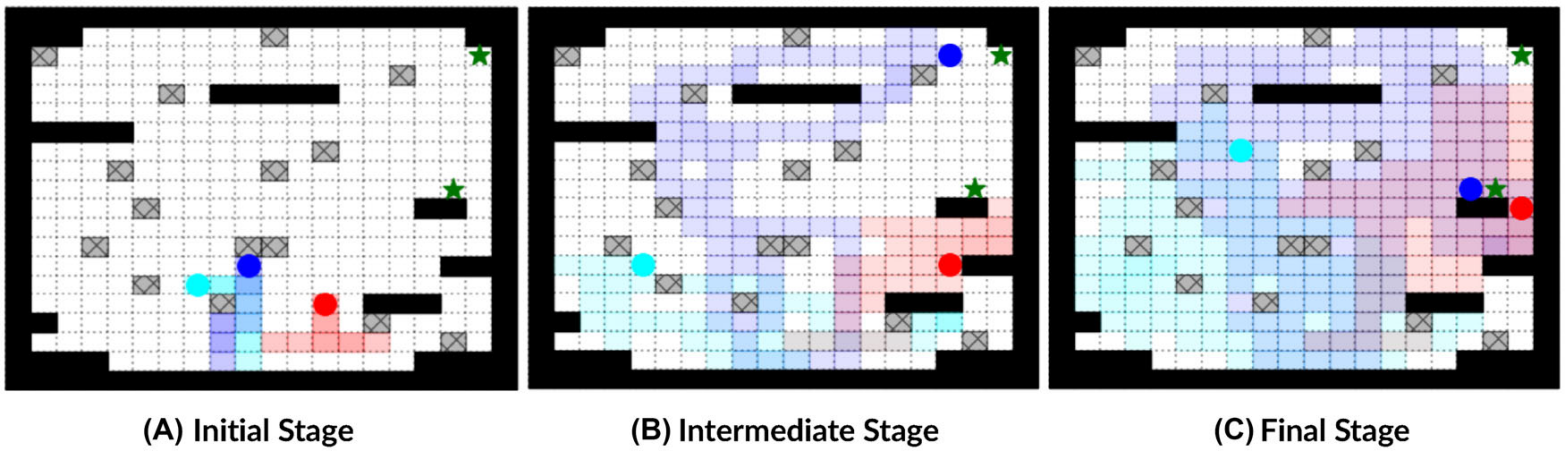
Agent trajectories in episodic progress stages for a sample scenario 4 simulation. (A) Initial stage: agents scatter to cover the grid while learning to avoid pitfalls. (B) Intermediate stage: agents have covered a fraction of the grid; adversarial agent misguides cooperative agents by focusing on only one part of the grid; agent trajectories remain separate. (C) Final stage: cooperative agents counter adversarial interference while retrieving targets in adversarial agent's area.

To visualize how our risk‐aware MARL algorithm mitigates coupled environmental and adversarial risks, Figure 6 displays cooperative and adversarial agent trajectories during different stages of an episode in scenario 4. At the initial stage (see Figure 6A), all agents move in different cardinal directions and try to cover the portion of the grid in their respective directions. We observe that both cooperative and adversarial agents learn to avoid pitfalls. At the intermediate stage (see Figure 6B), all agents traverse fractions of the grid in their respective directions. Since an adversarial agent trains to be noncooperative, the agent continues to repeatedly explore a relatively limited fraction of grid cells in the southeast region of the grid. Since each agent can detect another agent within three grid cells, the cooperative agents follow trajectories that do not significantly overlap the adversarial agent's trajectory. At the terminal stage (see Figure 6C), we observe that one of the cooperative agents traverses grid cells previously covered by the adversarial agent, indicating that the cooperative team learns to mitigate adversarial interference.

Based on our methodology and experimental results above, below are the key overarching insights for addressing risk‐aware autonomous SAR navigation challenges:

- POMDP provides a strong mathematical foundation for addressing autonomous SAR navigation challenges for risk‐informed decision support.
- Our proposed risk‐aware MARL algorithm, including state estimation with local awareness and composite reward shaping, when trained with coupled environmental and adversarial risks performs better than other methods in SAR navigation settings, particularly with respect to success rate.
- CTDE is an effective learning strategy for operationalizing the risk‐aware MARL algorithm and yields agents that are less susceptible to sudden changes due to environmental or adversarial factors.
- With risk‐aware MARL algorithm, both simulation performance metrics (*retrieval time* and *success rate*) yield intuitive trends, where increasing scenario complexity leads to higher retrieval times and lower success rates.

### 5.4 Real‐world implications

The proof‐of‐concept results in Section 5.3 demonstrate the effectiveness of the risk‐aware MARL algorithm to perform a SAR task under both environmental and adversarial risks. The policy of each navigation agent was trained in a CTDE setting using a generic gridworld topography (i.e., a navigable grid with a given configuration of target, pitfall and obstacle locations). As such, each agent learns a policy (i.e., a mapping from local state information to optimal action) that can be deployed to a new topography, without the need to train from scratch. Factors influencing this generalizability property of the risk‐aware MARL algorithm include: (i) training of each agent's policy on not only its own trajectories, but also trajectories of other agents in a collaborative team—thereby allowing the policy to be informed by state transitions that the agent may not have encountered itself, and (ii) decentralized policy of each agent being contingent on local state information (i.e., information about the grid cell that an agent occupies at a given time as well as the neighboring cells adjacent to the occupied grid cell) and not on detailed topographical information about the entire map. The agents can be trained in a controlled setting with a generic map ahead of deployment/testing in an environment that the agents may not have encountered in training. Therefore, this algorithm can potentially be applied to different real‐world navigation tasks where a team of collaborative autonomous agents are deployed, and where multiple sources of risk can arise from the environment as well as an active adversary. Real‐world examples of such navigation tasks include (but are not limited to) SAR (Yamauchi, 1997), emergency disaster management (Casper & Murphy, 2003), and surveillance (Witwicki et al., 2017). These tasks can involve a team of autonomous agents such as UAVs, USVs, or UGVs. Adversarial attacks on unmanned vehicles, especially those operated using machine learning and RL algorithms are increasingly gaining traction (Hickling et al., 2023; Tian et al., 2021) in the research community, and as such, it is critical to develop algorithms to mitigate these attacks. The environment where a navigation task is being performed may also pose additional risks, depending on the task. For instance, autonomous agents performing SAR in a postdisaster urban environment may encounter extreme voids or debris, which may damage the agent's structural health.

While this paper demonstrates that coupled impacts of adversarial and environmental risks can be mitigated using the risk‐aware MARL algorithm, several future steps still need to be taken before implementing such an algorithm in a real‐world setting. MARL (without the presence of an active adversary) for navigation of unmanned vehicles are being investigated recently in 3D simulation environment (e.g., AsirSim (Shah et al., 2018), Gazebo (Takaya et al., 2016)) and physical experimental testbeds for aerial navigation (Lou et al., 2023). Please note that experimentation with the gridworld abstraction in this paper is a “proof‐of‐concept” to demonstrate effectiveness of the proposed risk‐aware MARL algorithm. Improved performance (in terms of retrieval time and success rate) of a risk‐aware MARL agent in a gridworld setting over other approaches (Iqbal & Sha, 2019; Rahman et al., 2022) represents a step toward practical feasibility in more complex environments. However, application of this algorithm in a 3D simulation environment and further in a real‐world setting will require updates to state and action spaces based on additional complexity in the environment. It should be noted that our proposed algorithm is agnostic to specific state and action spaces and/or environmental specifications; and as such, can be adapted for more complex realistic environments. Additional complexity in the environment will lead to higher computational cost of training and testing. Parallel computation architectures and transfer learning schemes may help manage such compute cost challenges. Experimentation in 3D simulation environments, such as AirSim (Shah et al., 2018) and Gazebo (Takaya et al., 2016), is a planned future research step. Subsequent research with practical relevance can also focus on modeling communication technologies that allow information exchange between a team of unmanned vehicles (Sharma et al., 2020) as part of such 3D simulators, and investigate how risk‐aware MARL can mitigate adversarial attacks such as denial of service or man‐in‐the‐middle attacks. Such research endeavors can also investigate how human operators can infer adversarial intent to a specific attack—which can potentially be done by integrating the risk‐aware MARL algorithm with a hierarchical architecture including a human‐in‐the‐loop (Chatterjee et al., 2023).

## 6 CONCLUDING REMARKS

Sources of risk in autonomous SAR missions may emerge from uncertain environmental and adversarial factors. Multiagent robotic systems are promising in such settings, however, these systems need to incorporate risks from multiple sources in their learning strategies. We first presented a mathematically well‐grounded POMDP formulation based on a risk framework, that provides a sound conceptual foundation, for the multiagent autonomous SAR navigation problem. Thereafter, we quantified the coupled impact of environmental and adversarial risks, and subsequently developed a state estimation and composite reward‐shaping strategy used as part of a risk‐aware MARL algorithm to mitigate these risks. We observed that coupled environmental and adversarial risks lead to an increase in target retrieval time, based on risk‐aware MARL algorithm, by 47% (average) and 41% (median) when compared to a scenario with no risks. The proposed state estimation with local awareness and composite reward‐shaping strategy in our risk‐aware MARL algorithm, implemented with a CTDE strategy, improves the performance of autonomous agents in simulation settings with multiple risks (quantified in terms of average and median retrieval times) by 11%–23% and 18%– 29%, respectively, compared to the state‐of‐the‐art methods. However, the reduction in retrieval times is only statistically significant (based on a one‐sided K–S test at 95% confidence level) for the scenario where both adversarial and environmental risks are present, when risk‐aware MARL algorithm is compared against MARL with environmental risks. Furthermore, time and resource constraints significantly impact SAR operations, where the proposed risk‐aware MARL method performs better than other approaches in success rate under limited operational time, especially in high‐risk scenarios; risk‐aware MARL algorithm improves success rate by a margin of 20%–43% (i.e., an improvement by a factor >1.5) with a relatively significant computational cost, compared to the state‐of‐the‐art methods. The risk‐aware MARL algorithm can be generalized for deployment to new navigation environments due to the learned policy of each agent being informed by trajectories of all agents and being contingent on local state information. Future work may include advancing the proposed risk‐aware MARL algorithm to account for adversary detection, adapting the algorithmic strategies for mixed cooperative‐competitive environments with hierarchical teams of agents, and implementation of risk‐aware MARL algorithms in real‐world high‐fidelity simulation environments.

## ORCID

*Samrat Chatterjee*, https://orcid.org/0000‐0003‐1406‐1093

## Data Availability

The data that support the findings of this study are available from the corresponding author upon reasonable request.

## References

[risa70067-bib-0001] Amari, S.‐i. (1993). Backpropagation and stochastic gradient descent method. Neurocomputing, 5(4–5), 185–196.

[risa70067-bib-0002] Apostolakis, G. (1990). The concept of probability in safety assessments of technological systems. Science, 250(4986), 1359–1364. 2255906 10.1126/science.2255906

[risa70067-bib-0003] Apostolakis, G. E. (2004). How useful is quantitative risk assessment? Risk Analysis, 24(3), 515–520. 15209926 10.1111/j.0272-4332.2004.00455.x

[risa70067-bib-0004] Audigier, M. A. , Kiremidjian, A. S. , Chiu, S. S. , & King, S. A. (2000). Risk analysis of port facilities. In 12th World Conference on Earthquake Engineering (pp. 1–8).

[risa70067-bib-0005] Aven, T. (2012). The risk concept‐historical and recent development trends. Reliability Engineering & System Safety, 99, 33–44.

[risa70067-bib-0006] Aven, T. (2016). Risk assessment and risk management: Review of recent advances on their foundation. European Journal of Operational Research, 253(1), 1–13.

[risa70067-bib-0007] Baker, J. W. , & Allin Cornell, C. (2005). A vector‐valued ground motion intensity measure consisting of spectral acceleration and epsilon. Earthquake Engineering & Structural Dynamics, 34(10), 1193–1217.

[risa70067-bib-0008] Balaji, P. G. , & Srinivasan, D. (2010). An introduction to multi‐agent systems. In Innovations in multi‐agent systems and applications‐1 (pp. 1–27). Springer.

[risa70067-bib-0009] Banas, J. , Gray, A. , Goron, G. , Roberts, W. , Shah, S. , Zanella, P. , Griendling, K. , & Mavris, D. (2016). Determining robust control methods for coordinated UAVs in varying mission environments. https://dspace‐erf.nlr.nl/server/api/core/bitstreams/dceb72da‐d7dc‐4804‐991a‐738bf7fbd15a/content

[risa70067-bib-0010] Bier, V. M. , & Lin, S.‐W. (2013). On the treatment of uncertainty and variability in making decisions about risk. Risk Analysis, 33(10), 1899–1907. 23718895 10.1111/risa.12071

[risa70067-bib-0011] Casper, J. , & Murphy, R. R. (2003). Human‐robot interactions during the robot‐assisted urban search and rescue response at the world trade center. IEEE Transactions on Systems, Man, and Cybernetics, Part B (Cybernetics), 33(3), 367–385. 10.1109/TSMCB.2003.811794 18238185

[risa70067-bib-0012] Chatterjee, S. , Bhattacharya, A. , Dutta, A. , Rahman, A. , Ramachandran, T. , Chikkagoudar, S. , & Bharadwaj, R. (2023). Collaboration and negotiation. In Autonomous intelligent cyber defense agent (AICA): A comprehensive guide (pp. 229–251). Springer.

[risa70067-bib-0013] Cox, L. A., Jr (2020). Answerable and unanswerable questions in risk analysis with open‐world novelty. Risk Analysis, 40(S1), 2144–2177. 33000494 10.1111/risa.13553

[risa70067-bib-0014] Doroodgar, B. , Liu, Y. , & Nejat, G. (2014). A learning‐based semi‐autonomous controller for robotic exploration of unknown disaster scenes while searching for victims. IEEE Transactions on Cybernetics, 44(12), 2719–2732. 24760949 10.1109/TCYB.2014.2314294

[risa70067-bib-0015] Du, Y. , Chatterjee, S. , Bhattacharya, A. , Dutta, A. , & Halappanavar, M. (2023). Role of reinforcement learning for risk‐based robust control of cyber‐physical energy systems. Risk Analysis, 43(11), 2280–2297. 36746175 10.1111/risa.14104

[risa70067-bib-0016] Goel, S. , Tatiya, G. , Scheutz, M. , & Sinapov, J. (2021). NovelGridworlds: A benchmark environment for detecting and adapting to novelties in open worlds. In AAMAS Adaptive Learning Agents (ALA) Workshop .

[risa70067-bib-0017] Gonzalez, G. , Angulo, C. , & Raya, C. (2007). A multi‐agent‐based management approach for self‐health awareness in autonomous systems. In Fourth IEEE International Workshop on Engineering of Autonomic and Autonomous Systems (EASe '07) (pp. 79–88). IEEE.

[risa70067-bib-0018] Haarnoja, T. , Zhou, A. , Abbeel, P. , & Levine, S. (2018). Soft actor‐critic: Off‐policy maximum entropy deep reinforcement learning with a stochastic actor. In International Conference on Machine Learning (pp. 1861–1870). PMLR.

[risa70067-bib-0019] Haimes, Y. Y. (2009). On the definition of resilience in systems. Risk Analysis, 29(4), 498–501. 19335545 10.1111/j.1539-6924.2009.01216.x

[risa70067-bib-0020] He, W. , Xu, W. , Ge, X. , Han, Q.‐L. , Du, W. , & Qian, F. (2021). Secure control of multiagent systems against malicious attacks: A brief survey. IEEE Transactions on Industrial Informatics, 18(6), 3595–3608.

[risa70067-bib-0021] Hickling, T. , Aouf, N. , & Spencer, P. (2023). Robust adversarial attacks detection based on explainable deep reinforcement learning for UAV guidance and planning. IEEE Transactions on Intelligent Vehicles, 8(10), 4381–4394.

[risa70067-bib-0022] Hodges, J. L., Jr (1958). The significance probability of the Smirnov two‐sample test. Arkiv för Matematik, 3(5), 469–486.

[risa70067-bib-0023] Ikeda, T. , & Shibuya, T. (2022). Centralized training with decentralized execution reinforcement learning for cooperative multi‐agent systems with communication delay. In 61st Annual Conference of the Society of Instrument and Control Engineers (SICE) (pp. 135–140). IEEE.

[risa70067-bib-0024] Iqbal, S. , & Sha, F. (2019). Coordinated exploration via intrinsic rewards for multi‐agent reinforcement learning. *arXiv preprint arXiv:1905.12127* .

[risa70067-bib-0025] Kaelbling, L. P. , Littman, M. L. , & Moore, A. W. (1996). Reinforcement learning: A survey. Journal of Artificial Intelligence Research, 4, 237–285.

[risa70067-bib-0026] Kaplan, S. , & Garrick, B. J. (1981). On the quantitative definition of risk. Risk Analysis, 1(1), 11–27. 10.1111/0272-4332.215153 11798118

[risa70067-bib-0027] Kingma, D. , & Ba, J. (2015). Adam: A method for stochastic optimization. In International Conference on Learning Representations (ICLR) 2015, San Diego, CA, USA (pp. 1–15).

[risa70067-bib-0028] Kochenderfer, M. J. (2015). Decision making under uncertainty: Theory and application. MIT Press.

[risa70067-bib-0029] Lee, H.‐R. , & Lee, T. (2021). Multi‐agent reinforcement learning algorithm to solve a partially‐observable multi‐agent problem in disaster response. European Journal of Operational Research, 291(1), 296–308.

[risa70067-bib-0030] Li, X.‐Y. , & Wang, X. (2025). Rescue path planning for urban flood: A deep reinforcement learning‐based approach. Risk Analysis, 45(4), 928–943. 39128862 10.1111/risa.17599

[risa70067-bib-0031] Lou, J. , Wu, W. , Liao, S. , & Shi, R. (2023). Air‐M: A visual reality many‐agent reinforcement learning platform for large‐scale aerial unmanned system. In IEEE/RSJ International Conference on Intelligent Robots and Systems (IROS) (pp. 5598–5605).

[risa70067-bib-0032] Mei, Y. , Lu, Y.‐H. , Lee, C. G. , & Hu, Y. C. (2006). Energy‐efficient mobile robot exploration. In Proceedings of the IEEE International Conference on Robotics and Automation, 2006, ICRA 2006 (pp. 505–511). IEEE.

[risa70067-bib-0033] Moehle, J. , & Deierlein, G. G. (2004). A framework methodology for performance‐based earthquake engineering. In 13th World Conference on Earthquake Engineering , Vol. 679 (12pp). WCEE Vancouver.

[risa70067-bib-0034] Nathan, P. T. , Almurib, H. A. , & Kumar, T. N. (2011). A review of autonomous multi‐agent quad‐rotor control techniques and applications. In 4th International Conference on Mechatronics (ICOM) (pp. 1–7). IEEE.

[risa70067-bib-0035] Ota, J. (2006). Multi‐agent robot systems as distributed autonomous systems. Advanced Engineering Informatics, 20(1), 59–70.

[risa70067-bib-0036] Paté‐Cornell, M. E. (1996). Uncertainties in risk analysis: Six levels of treatment. Reliability Engineering & System Safety, 54(2‐3), 95–111.

[risa70067-bib-0037] Porter, K. A. (2003). An overview of peer's performance‐based earthquake engineering methodology. In Proceedings of Ninth International Conference on Applications of Statistics and Probability in Civil Engineering (pp. 1–8).

[risa70067-bib-0038] Queralta, J. P. , Taipalmaa, J. , Pullinen, B. C. , Sarker, V. K. , Gia, T. N. , Tenhunen, H. , Gabbouj, M. , Raitoharju, J. , & Westerlund, T. (2020). Collaborative multi‐robot search and rescue: Planning, coordination, perception, and active vision. IEEE Access, 8, 191617–191643.

[risa70067-bib-0039] Rahman, A. , Bhattacharya, A. , Ramachandran, T. , Mukherjee, S. , Sharma, H. , Fujimoto, T. , & Chatterjee, S. (2022). AdverSAR: Adversarial search and rescue via multi‐agent reinforcement learning. In 2022 IEEE International Symposium on Technologies for Homeland Security (HST) (pp. 1–7). IEEE.

[risa70067-bib-0040] Russell, S. J. , & Norvig, P. (2016). Artificial intelligence: A modern approach. Pearson.

[risa70067-bib-0041] Shah, S. , Dey, D. , Lovett, C. , & Kapoor, A. (2018). AirSim: High‐fidelity visual and physical simulation for autonomous vehicles. In Field and Service Robotics: Results of the 11th International Conference (pp. 621–635). Springer.

[risa70067-bib-0042] Sharma, A. , Vanjani, P. , Paliwal, N. , Basnayaka, C. M. W. , Jayakody, D. N. K. , Wang, H.‐C. , & Muthuchidambaranathan, P. (2020). Communication and networking technologies for UAVs: A survey. Journal of Network and Computer Applications, 168, 102739.

[risa70067-bib-0043] Skoglund, C. (2021). Risk‐aware autonomous driving using POMDPS and responsibility‐sensitive safety. https://www.diva‐portal.org/smash/get/diva2:1590529/FULLTEXT01.pdf

[risa70067-bib-0044] Society for Risk Analysis . (2018a). Society for risk analysis glossary . https://www.sra.org/wp‐content/uploads/2020/04/SRAGlossary‐FINAL.pdf (accessed: 03.05.2024)

[risa70067-bib-0045] Society for Risk Analysis . (2018b). Risk analysis: Fundamental principles . https://www.sra.org/wp‐content/uploads/2020/04/SRA‐Fundamental‐Principles‐R2.pdf (accessed: 03.05.2024)

[risa70067-bib-0046] Srinivasan, S. , Lanctot, M. , Zambaldi, V. , Pérolat, J. , Tuyls, K. , Munos, R. , & Bowling, M. (2018). Actor‐critic policy optimization in partially observable multiagent environments. In Advances in neural information processing systems , 31.

[risa70067-bib-0047] Sung, Y. (2019). Multi‐robot coordination for hazardous environmental monitoring [PhD thesis, Virginia Tech].

[risa70067-bib-0048] Sutton, R. S. , & Barto, A. G. (2018). Reinforcement learning: An introduction. MIT press.

[risa70067-bib-0049] Takaya, K. , Asai, T. , Kroumov, V. , & Smarandache, F. (2016). Simulation environment for mobile robots testing using ROS and Gazebo. In 2016 20th International Conference on System Theory, Control and Computing (ICSTCC) (pp. 96–101).

[risa70067-bib-0050] Tian, J. , Wang, B. , Guo, R. , Wang, Z. , Cao, K. , & Wang, X. (2021). Adversarial attacks and defenses for deep‐learning‐based unmanned aerial vehicles. IEEE Internet of Things Journal, 9(22), 22399–22409.

[risa70067-bib-0051] Witwicki, S. , Castillo, J. C. , Messias, J. , Capitan, J. , Melo, F. S. , Lima, P. U. , & Veloso, M. (2017). Autonomous surveillance robots: A decision‐making framework for networked muiltiagent systems. IEEE Robotics & Automation Magazine, 24(3), 52–64.

[risa70067-bib-0052] Yamauchi, B. (1997). A frontier‐based approach for autonomous exploration. In Proceedings of the IEEE International Symposium on Computational Intelligence in Robotics and Automation CIRA'97. “Towards New Computational Principles for Robotics and Automation” (pp. 146–151). IEEE.

